# Validation of the historical adulthood physical activity questionnaire (HAPAQ) against objective measurements of physical activity

**DOI:** 10.1186/1479-5868-7-54

**Published:** 2010-06-24

**Authors:** Hervé Besson, Ceryl A Harwood, Ulf Ekelund, Francis M Finucane, Christopher J McDermott, Pamela J Shaw, Nicholas J Wareham

**Affiliations:** 1 Medical Research Council Epidemiology Unit, IMS, Cambridge, UK; 2 Academic Neurology Unit, Department of Neuroscience, University of Sheffield, UK

## Abstract

**Background:**

Lifetime physical activity energy expenditure (PAEE) is an important determinant of risk for many chronic diseases but remains challenging to measure. Previously reported historical physical activity (PA) questionnaires appear to be reliable, but their validity is less well established.

**Methods:**

We sought to design and validate an historical adulthood PA questionnaire (HAPAQ) against objective PA measurements from the same individuals. We recruited from a population-based cohort in Cambridgeshire, UK, (Medical Research Council Ely Study) in whom PA measurements, using individually calibrated heart rate monitoring, had been obtained in the past, once between 1994 and 1996 and once between 2000 and 2002. 100 individuals from this cohort attended for interview. Historical PA within the domains of home, work, transport, sport and exercise was recalled using the questionnaire by asking closed questions repeated for several discrete time periods from the age of 20 years old to their current age. The average PAEE from the 2 periods of objective measurements was compared to the self-reported data from the corresponding time periods in the questionnaire.

**Results:**

Significant correlations were observed between HAPAQ-derived and objectively measured total PAEE for both time periods (Spearman r = 0.44; P < 0.001). Similarly, self-reported time spent in vigorous PA was significantly correlated with objective measurements of vigorous PA (Spearman r = 0.40; P < 0.001).

**Conclusions:**

HAPAQ demonstrates convergent validity for total PAEE and vigorous PA. This instrument will be useful for ranking individuals according to their past PA in studies of chronic disease aetiology, where activity may be an important underlying factor contributing to disease pathogenesis.

## Introduction

Physical activity energy expenditure (PAEE) describes the energy used to perform all activities undertaken in daily living in excess of an individual's resting metabolic rate. Cumulative PAEE during a person's lifetime is thought to play an important role in determining the risk of developing several chronic diseases in later life [1-4]. In an aging population with an increasingly sedentary lifestyle, the public health impact of physical inactivity is likely to be substantial. Considering this, a valid and practical tool for measuring total historical physical activity (PA) may have many potential applications, including quantifying disease risk and determining the role of physical activity in the pathogenesis of diseases with long latency periods. However, PAEE is notoriously difficult to measure in free-living situations. Retrospective measurement of historical PAEE poses an even greater challenge, given the difficulty in validating such measurements.

Questionnaires provide a practical data collection tool for use in population-based studies. Although several PA questionnaires exist, most consider the PA undertaken over short time frames of days to months and often only within specific domains of life, such as occupation. Only a small number of questionnaires, considered historical, collect data regarding total PA over periods longer than one year. Although the reproducibility of several of these historical PA questionnaires has been demonstrated [5,6], the validity of such questionnaires is less well established. Some studies have compared data from contemporary questionnaires with those from similar questionnaires administered previously [7-11]. An important limitation of this approach is the subjective nature of the criterion variable, such that any correlation demonstrated may reflect correlated errors rather than the true validity of the questionnaire. Very few historical PA questionnaires have been validated against objective measurements of PA. One study confirmed modest validity (r = 0.29) of a questionnaire measuring PA up to 5 years ago when compared with uniaxial accelerometer measurements from the same time [12]. The validity of historical PA questionnaires against repeated objective measures of PAEE has not previously been determined.

As part of the Medical Research Council (MRC) Ely Study (Cambridgeshire, UK), members of a population-based cohort underwent objective PAEE measurements twice in the past [13]. This provided a unique opportunity to validate self-reported historical PA data against objective measurements of PAEE in the same individuals. We therefore sought to design and determine the validity of a novel, interviewer administered historical adulthood PA questionnaire (HAPAQ) against objective PAEE data collected up to 15 years ago.

## Methods

### Study participants

**Figure 1:**
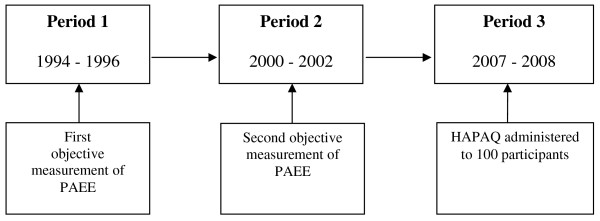
**Study timeline**. Objective measures of physical activity energy expenditure and delivery of HAPAQ.

Participants were recruited from the MRC Ely Study [14,15], a population-based prospective cohort study of the aetiology of type 2 diabetes and other metabolic disorders. The current mean age of the cohort is 65 years. Objective measurements of PAEE were taken from 394 members of this cohort at two separate time points, once between 1994 and 1996 (period 1) and again between 2000 and 2002 (period 2) (Figure 1). Of these, in December 2007, 197 individuals were ineligible for this study as they had previously declined further research participation, were currently recruited to another study or had died. Invitations were sent to the remaining individuals, of which 108 agreed to participate (55%). 100 attended for interview between December 2007 and March 2008. Ethical approval for the study was granted by the Cambridge Local Research Ethics Committee and all participants provided written informed consent.

### Objective measurement of physical activity

Objective measurements of PAEE were obtained over four consecutive days during periods 1 and 2 using minute-by-minute heart rate (HR) monitoring with individual calibration [16]. The relationship between oxygen consumption and HR was determined for each individual by measuring expired oxygen concentration, ventilation and HR following 10 minutes of supine rest and during four stages of graded exercise intensity. A cycle ergometer was used during period 1 and a treadmill was used during period 2. Energy expenditure (kJ min^-1^) at each exercise intensity was calculated from oxygen consumption data. Subsequently, HR monitors were worn during the waking hours over a 4 day period. The resulting HR measurements were used to derive estimates of PAEE during that period according to the flex HR method, so as to adjust for the reduced accuracy of HR monitoring when estimating lower intensity PA [17,18]. The flex HR was determined as the mean of the highest resting HR and lowest exercising HR measured during the fitness test [16]. For each minute the free-living measured HR exceeded the flex HR, the individual calibration data for the HR-oxygen consumption uptake were used to calculate PAEE. The percentage of time spent in vigorous PA (VPA) (≥ 6.5 METs) was calculated as the proportion of time during which HR was greater than 1.75 times the resting HR, guided by previous work [19].

### Questionnaire design

HAPAQ was designed to collect data regarding total regular PA undertaken from the age of 20 years to their current age (see additional file 1). To do this, the questionnaire was divided into discrete time periods, starting with the most recent 15 years in three 5-year sections. Following this, questions regarding PA from the age of 20 years until the most recent 15 years were asked in 10-year sections. For each section, an identical set of closed questions were asked about PA in the domains of home, work, transport, sport (defined as strenuous sporting activities which make you breathless or cause noticeable sweating) and exercise (defined as less strenuous leisure activities). The nature, duration and frequency of regular activities recalled by the participant for each time period were recorded. Occupational activity was categorized according to the Modified Tecumseh Occupational Activity Questionnaire [20,21]. Although participation in gardening, home and car maintenance (referred to as do it yourself or "DIY") and housework was documented, the durations of these activities were not as guided by previous work suggesting that recall of durations of unstructured activities correlates poorly with objective measurement of total PA [22,23]. To improve question comprehension, flash cards were used to depict descriptive terms in a visual format. The questionnaire was designed in an electronic format to aid delivery and data management.

### The HAPAQ interview

The questionnaire was delivered through face-to-face interviews to avoid interpretation errors, reduce incomplete data collection and to allow the application of cognitive interview techniques to improve recall [24]. All interviews were conducted by the same interviewer (CAH), with a maximum of 90 minutes allocated per interview. A life calendar, documenting the dates of important life events for the individual, such as marriage and child birth, was completed prior to the questionnaire, in order to aid recall.

### Data reduction from the questionnaire

Subjective measurements of total PAEE were derived from questionnaire data to allow direct comparison with the objective PA measurements. Each reported activity was allocated an energy expenditure score as a measure of intensity, expressed in METs, as guided by the Compendium of Physical Activities [25,26]. For those sections of the questionnaire that corresponded to the years of the objective PAEE measurements, durations of each reported activity were multiplied by the intensity of that activity to provide energy expenditure (EE) scores in MET hours-day. As durations were not collected for household activities, the following daily durations were assigned: 1 hour for housework, 0.14 hours for DIY (equivalent to one hour per week) and 0.10 hours for gardening (equivalent to one hour per week within 8 months of the year). The duration for commuting was calculated by dividing the reported return journey distance by an allocated speed of 3 miles per hour for walking and 10 miles per hour for cycling, and then multiplying this by the number of return journeys per week. This figure was then multiplied by 0.9 as it was assumed that individuals are on holiday for 10% of the weeks of the year. Energy expenditure for each activity was weighted by the number of years the given activity was reported divided by the number of years the period encompasses. Given that 1 MET is equivalent to approximately 3.5 ml kg^-1 ^min^-1 ^oxygen consumption and that the energy equivalent of one litre of oxygen is considered to be 20.3 kJ [27], daily EE scores were converted to PAEE (kJ/min) using the following equation:

Guided by previous observations that reported housework was inversely related to PAEE [22,23] subjective measurements of housework were not used for the construction of this score.

The percentage of time spent in VPA, defined as activities with a MET score ≥ 6.5, was also calculated for each participant.

The following assumptions were made when deriving PAEE variables from self-report data. All participants were allocated the same duration of sleep of 8 hours. This approach was chosen as recall of sleep duration has been previously reported as inaccurate [28], a problem likely to be exacerbated by the historical nature of this questionnaire. When 16 to 18 hours of daily activity were reported, the sleeping time was reduced proportionally. If more than 18 hours per day were reported, the duration of each activity or inactivity (hrs/day) was scaled down proportionally to adjust for this. When an individual reported fewer than sixteen hours per day, the time unaccounted for was assigned a predetermined intensity level of 1.5 METs, chosen as low intensity activities have been reported as poorly recalled by PA questionnaires [21]. This MET score is considered to be the threshold between sedentary and light activities [25,26]. This approach ensured that HAPAQ-derived PAEE was calculated for the same number of hours as the objective measurements of PA.

### Statistical analysis

Self-reported and objective PA measurements were compared between the 2 periods using a paired t-test for continuous variables and a McNemar's test for categorical variables. Spearman's correlation coefficients and Intraclass correlation coefficients (ICC) were used to determine any associations between the objective measurements of PA and the corresponding questionnaire-derived PA measurements. ICC were calculated using log-transformed variables and zero values were converted to the value 10^-9^. The interaction term "self-reported physical activity x period" was also included in the rank regression model to test the heterogeneity of the correlation by periods. This heterogeneity was not significant for PAEE (p = 0.64) or for VPA (p = 0.59). Therefore, the average of the PA variables from periods 1 and 2 were calculated from the self-reported and objective measurements, referred to as merged data, and used for the subsequent analyses.

Multiple rank regression analyses were performed to assess Spearman partial correlation between objective PAEE and self-reported PAEE, adjusting for body weight. The interaction terms "self-reported physical activity × gender", "self-reported physical activity × age" and "self-reported physical activity × body mass index (BMI)" were also included in preliminary models. The agreement between self-report and objective measurements of PAEE and VPA was assessed using a modified Bland-Altman technique. For each Bland-Altman plot, the x-axis represents the objective PA measurements and the y-axis the difference between questionnaire-derived measurements and objective measurements. Mean bias was defined as the average of the difference between objectively and subjectively measured PAEE. A relative bias was also determined as the ratio between the objective and subjective measures of PA. The correlation of the objective PA measurements with both the difference between the objective and subjective PA and the ratio of the objective and subjective measures of PA was calculated, to give the proportional errors of these two biases. The limits of agreement were set at two standard deviations above and below the mean bias, as described previously [29,30].

A categorical PAEE index was also constructed from the merged HAPAQ-derived data. This index was based on quartiles of EE (kJ/min) above resting EE as follows: Inactive (< 3.5 kJ/min); Moderately inactive (3.5 to 4.5 kJ/min); Moderately active (> 4.5 to 6 kJ/min); Active (> 6 kJ/min). VPA was divided into three categories: 0% of time spent in VPA, > 0%-1.3% of time spent in VPA and > 1.3% time spent in VPA. The first category consisted of half of the total participants. The other half of the participants was divided into two categories based on the median of reported VPA. These same cut-offs were use to categorize the corresponding objective measurements of PAEE and VPA. Agreements between these PA indices and their corresponding categorized objective measurements were assessed using Cohen's weighted Kappa. Analyses were conducted using Statistical Analysis System software version 9.1 [31].

## Results

In total, 44 men and 56 women attended for interview, which lasted on average 66 minutes. Men were slightly older than women (mean age 65.7 ± 5.0 versus 63.5 ± 3.9 years) and were significantly more physically active according to objective and self- reported measurements of total PAEE (data not shown). The characteristics and PA measurements of the study participants are summarized by periods in Table 1. No significant difference was observed for the percentage of time spent in VPA between periods 1 and 2, regardless of measurement method. In contrast, objectively measured total PAEE was significantly higher during the second period compared to the first (p < 0.0001), whereas self- reported PAEE was significantly lower (p < 0.0001). Regarding domain specific PA measurements, PAEE at work was significantly higher in period 1 compared to period 2. There were no other statistically significant differences in any other variables between the two periods.

**Table 1:** Characteristics and physical activity measurements of participants recruited to the HAPAQ validation study

	Period 1 (1994 - 1996)		Period 2 (2000 - 2002)		Period comparison*
	Median	IQR	Median	IQR	*P *value
BMI (kg/m^2^)	25.3	4.2	26.0	4.8	0.31
**Objective measurements PA:**					
Proportion time spent in VPA (%)	0.6	2.2	0.7	2.5	0.15
Total PAEE (kJ/min)	5.8	3.6	7.3	5.9	< 0.0001
**HAPAQ- derived measures PA:**					
Proportion time spent in VPA (%)	0.0	1.3	0.0	1.3	0.27
PAEE at home (kJ/min)	0.5	0.4	0.5	0.4	0.25
PAEE at work (kJ/min)	2.1	2.1	1.6	1.9	0.0002
PAEE for transportation (kJ/min)	0.0	0.1	0.0	0.1	0.11
PAEE for sport (kJ/min)	0.0	0.1	0.0	0.1	0.96
PAEE for exercise (kJ/min)	0.5	0.7	0.5	0.8	0.22
Total reported PAEE (kJ/min)	3.1	3.0	2.7	2.5	0.0003
Proportion retired (%)	12%		28%		< 0.0001

* Paired t-test used for continuous variables and McNemar's test used for categorical variables

BMI: Body mass index

VPA: Vigorous Physical Activity

PAEE: Physical Activity Energy Expenditure

IQR: Interquartile range

Following merging the PA data from periods 1 and 2, the correlation coefficients and median biases between HAPAQ-derived estimates and objective measurements of PAEE are displayed in Table 2. The objective measurements of total PAEE correlated significantly with HAPAQ-derived measurements (r = 0.44, p < 0.0001), which remained unchanged after adjustment for the body weight. This correlation improved (r = 0.48, p < 0.0001) after correcting for time that was unaccounted for in the HAPAQ data, referred to as PAEE^b ^in table 2 (i.e. assigned a MET value of 1.5) and this corresponded to an ICC of 0.39 (p < 0.0001), which was not statistically significant before the correction.

**Table 2:** Validity of HAPAQ: HAPAQ-derived physical activity measurements, Spearman and intraclass correlations with objective physical activity measurements and the median biases from agreeement analysis

	HAPAQ-derived measurements		Spearman correlation	Intraclass correlation coefficient^c^	HAPAQ biases	
	Median	IQR			Median	IQR
Proportion time spent in VPA (%)	0.01	1.32	0.40***	0.24**	-0.26	1.59
Total reported PAEE (kJ/min)^a^	2.91	2.66	0.44***	0.01	-2.80	4.24
Total reported PAEE (kJ/min)^b^	5.90	2.28	0.48***	0.39***	-0.16	3.50

* *P *< 0.05

** *P *< 0.01

*** *P *< 0.0001

VPA: Vigorous physical activity

PAEE: Physical activity energy expenditure

IQR: Interquartile range

^a^excluding housework from PAEE

^b^as per ^a ^with remaining time designated as light activity (MET 1.5)

^c^Variables were log-transformed

Regarding domain specific PA (data not shown), the highest correlation with objective PAEE was observed for PAEE at work (r = 0.35; p = 0.0003) with the lowest correlation for PAEE in the home (r = -0.09; p = 0.34). Within household activities, housework was negatively correlated with objective measure of PAEE (r = -0.24; p = 0.01), supporting the findings of previous PA studies [22,23], whereas DIY and gardening were positively correlated with objectively measured PAEE.

**Figure 2:**
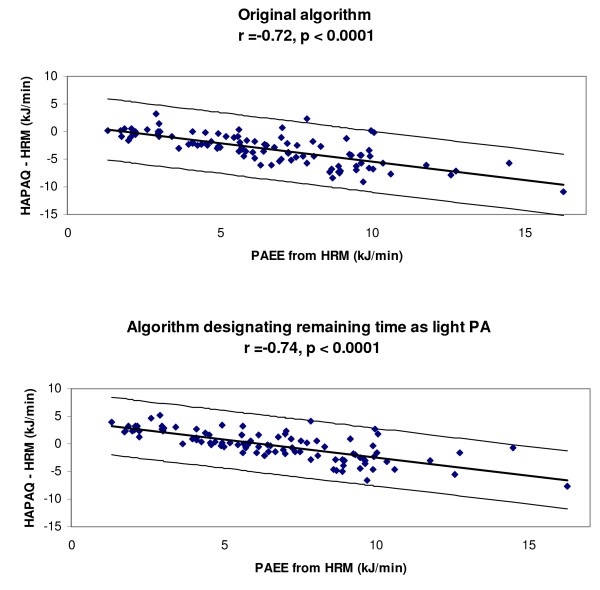
**Bland and Altman plots displaying the difference between objective and HAPAQ- derived measures of total PAEE against objective PAEE measure**. The correlation between the differences and objective PAEE measures is denoted "r".

The biases displayed in table 2 demonstrate that, in general, HAPAQ tended to underestimate total PAEE (median bias -2.80, IQR = 4.24). The underestimation was greatest for data recalled in period 2 (median bias -3.60, IQR = 6.48) compared to period 1 (median bias -1.69, IQR = 3.44). After correction for unaccounted time, the underestimation for the merged data was less (-0.16, IQR = 3.50). As shown in Figure 2, the mean bias between the two methods was proportional to the objectively measured PAEE. The relative bias was also significantly proportional to the objective PAEE measurement (data not shown).

**Figure 3:**
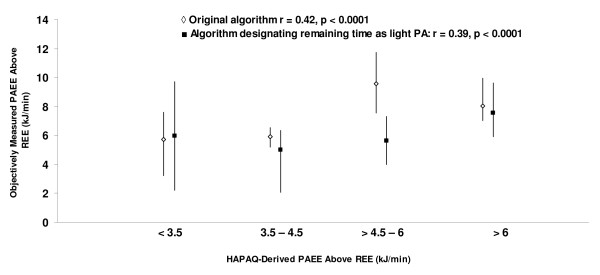
**Distribution of objective measures of PAEE according to the four levels of the HAPAQ PAEE index, expressed in kJ.min^-1^**. Data are expressed as medians and interquartile ranges.

When self-reported total PAEE was stratified into quartiles and compared with objectively measured PAEE, significant correlations were observed (Figure 3). The weighted Kappas were modest for both the original algorithm (0.16; p = 0.001) and when the remaining time was designated as light activity (0.22; p = 0.0008).

**Figure 4:**
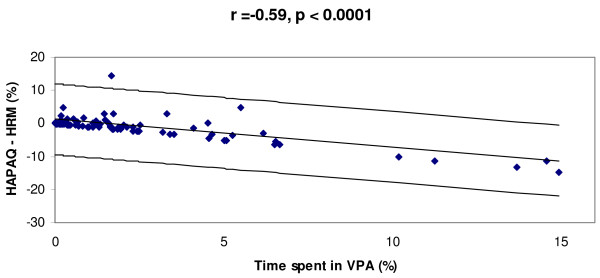
**Bland and Altman plots displaying the difference between objective and HAPAQ- derived measures of percentage time spent in VPA against objective VPA measures**. The correlation between the differences and objective VPA measurements is denoted "r".

**Figure 5:**
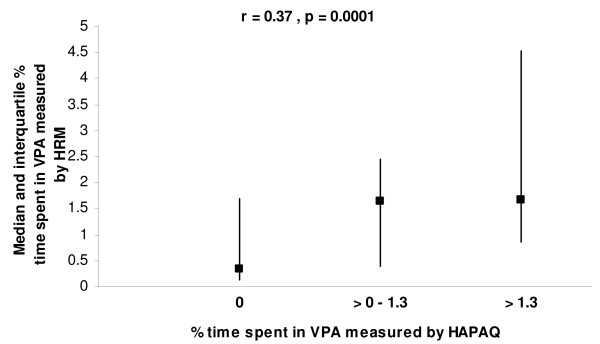
**Distribution of objective measurements of the percentage of time spent in vigorous physical activity (VPA) by the three levels of VPA measured using HAPAQ**. Data are expressed as medians and inter-quartile ranges.

Regarding time spent in VPA, estimations derived from HAPAQ correlated significantly with objective measurements (r = 0.40, p < 0.0001; ICC = 0.24, p = 0.008) (Table 2). HAPAQ tended to underestimate VPA (median bias -0.26, IQR = 1.59). The underestimation was very similar for periods 1 (median bias -0.21, IQR = 0.97) and period 2 (-0.16, IQR = 1.99). In figure 4, the Bland and Altman plots demonstrate that the mean bias between self-reported and objectively measured VPA is proportional to the objectively measured VPA, though the relative bias did not display the same significantly proportional error (data not shown). The distribution of objectively measured VPA by the self-reported VPA categories and the correlation between these measurements are shown in Figure 5. The weighted Kappa between categorized self-reported PA and the corresponding categorized objective measurement was very modest (0.13; p = 0.009).

## Discussion

To the best of our knowledge, this study is the first to validate a 15 year self-reported historical PA questionnaire against repeated objective measurements of PAEE measured in the same individuals in the past. Our results suggest that questionnaire-derived estimates of PA have acceptable convergent validity with objective PA measurements from the same time periods. Therefore, HAPAQ can be used to accurately rank individuals according to their level of PA in the past.

Fair correlations were observed between objective and self-reported measurements of time spent in VPA and total PAEE. These correlations are comparable with previous historical PA questionnaires studies, reporting correlations of 0.09 to 0.5 [7-11], though in contrast to our work these studies compared questionnaire data against other subjective PA measurements. The convergent validity results are also similar to those reported in a recent review of previous studies which examined the validity of self- reported PA against the objective measurement of doubly labeled water [32]. However, none of the questionnaires in this review quantified PA over a period of time longer than a year and therefore cannot be considered historical.

Strong correlations were observed between objective and self-reported PA measurements when the averages of the two time periods were compared. When studying periods 1 and 2 separately, these correlations remained significant and were comparable to those reported by previous PA questionnaires with shorter periods of recall [32]. The correlations between self-reported PA and objective PA measurements were confirmed when self-reported data were categorized, providing further support for this questionnaire's ability to reliably rank individuals according to total PAEE and time spent in VPA.

The agreement analyses demonstrate that overall HAPAQ tended to underestimate PA. On average, the daily time accounted for by the questionnaire data was 5.6 (+/-2.2) hours for period 1 and 6.2 (+/-2.0) hours for period 2, which would certainly help to explain this underestimation. However, although HAPAQ does appear to underestimate VPA in more active individuals, as demonstrated in figure 4, HAPAQ may accurately estimate the time spent in VPA in individuals who undertook very little vigorous activity. This discrepancy may be explained either by under-reporting of VPA by active individuals or insufficient questionnaire sensitivity to detect VPA.

When estimating total PAEE, HAPAQ underestimated PAEE in more active individuals (figure 2). However, in individuals with sedentary lifestyles, the questionnaire overestimated

total PAEE, particularly when adjusting for time unaccounted for by HAPAQ. Considering this, HAPAQ may have a tendency to underestimate associations between disease and an active lifestyle, while any positive association identified using the questionnaire is likely to be valid. As recall of light PA using questionnaires has previously been noted as problematic [21], possibly due to the unstructured and routine nature in which these activities are performed, this may help to explain the overestimation of PAEE in sedentary participants. Inactive individuals may feel compelled to overestimate their PA levels, particularly in view of the growing public health campaign to disseminate the health benefits of PA and to promote an active lifestyle. Limitations in the collection of subjective total PAEE arising from poor recall of light intensity activities are likely to be inherent to historical questionnaires, particularly when a sizeable proportion of total daily activities are sedentary [33,34]. However, collection of such data is imperative as associations with many chronic diseases prevalent in modern society are established. At present, historical PA questionnaires offer the most feasible and acceptable option for this measurement.

Although imprecision of the questionnaire-derived data may account for the apparent over- and underestimations of HAPAQ, the potential inaccuracy of the objective PA measurements may provide an alternative explanation. Estimation of an individual's habitual PA from two 4 days periods could misclassify individuals regarding their PA levels, depending on the activities undertaken in those 4 days.

A recent literature review illustrated that although the validity of existing PA questionnaires against objective PA measurements has been undertaken, most of the questionnaires involve short-term recall of PA data, with none collecting PA data from more than a year ago [32]. DuBose et al reported the only other study to examine the validity of a truly historical questionnaire against objective measurements, using accelerometer-derived data. However, the time lapse between collection of objective and self-reported PA measurements was only 3-5 years and the study population was restricted to women [12]. They reported a correlation of r = 0.29 between objective and self-report PA estimates, which is lower than in the present study. Furthermore, results on the validity of time spent at VPA were not available. Our study applies to both genders, whilst many previous historical questionnaire validation studies have been restricted to women [5,6,11,12].

HAPAQ was designed to allow the application of techniques to optimise accurate data collection. All questions in the HAPAQ were simple closed questions so as to minimize ambiguity [24]. The questionnaire was structured into domains of PA as long-term memory is known to encode PA within the context in which it was performed [35]. As duration of activity tends to be more difficult to recall [36], data were collected in a disaggregated manner, such that participants were asked first about activity type, then frequency and finally duration. To facilitate recall and help orientate participants, a life calendar was constructed and repeatedly referred to during the interview. Temporal memory cues were also positioned throughout the questionnaire [37].

When interpreting the results of this study, several limitations should be considered. The generalisability of our findings to different study populations may be questioned when using HAPAQ in future studies. Factors to consider include differences between participants and non-participants and the age distribution of the study population, though such an age group would be appropriate when studying disease association with PA for many important conditions with onset later in life. Although the questionnaire collects PA data for the whole of adulthood, we can only validate data up to 15 years ago due to the timing of the objective PA measurements. However, the same data collection methods were used throughout the questionnaire. Therefore, in view of the validity of data over the last 15 years, this may be an appropriate tool to estimate PA over timeframes extending beyond 15 years in the past. Our criterion method, (i.e. individually calibrated HR monitoring) is thought to be less accurate at estimating energy expenditure for low intensity activities [38]. The HR monitoring calibration protocols differed between periods 1 and 2, making comparisons of absolute PAEE measurements from these two periods difficult. However, as the heterogeneity of correlation by time period was not significant, it is doubtful that the different protocols would affect the ranking of individuals according to objectively measured PAEE. Therefore, the significant associations between the objectively measured and self-reported variables are likely to represent a true association. We compared subjective PAEE estimates from two 5-year periods with objective PAEE measurements obtained from two periods of only four days. Although this reduces the temporal accuracy of these comparisons, detailed recall from a focused time frame 15 years ago is unrealistic and may not be representative of habitual PA. Conversely, objective PA measurements over a considerably longer time period would be unfeasible. By merging the data from the two periods, as was done in this study, this provides some adjustment for the inevitable measurement error when quantifying a parameter with high within subject variance. An average of 8 days of objective measurements of PA taken over a 5 year period was thought to be more representative of habitual PA undertaken during a time frame of 10 years, and this reasoning is supported by previous work [39,40].

We acknowledge the limitations a 66 minute PA questionnaire may impose. However, this study demonstrates the feasibility of interviewing 100 individuals over a 3 month period. We received positive feedback from participants regarding the interview experience. In addition, HAPAQ only needs to be delivered once to collect adulthood PA data, rather than repeated visits required for other questionnaires. However, we do acknowledge that such a time commitment may deter certain individuals from participating (e.g., individuals in full time employment). Finally, the test-retest reliability of HAPAQ remains to be determined.

## Conclusions

This is the first study to demonstrate the validity of retrospectively self-reported PA undertaken up to 15 years ago against objectively measured PAEE from the same time period. The consistency of the results of our analyses from both time periods suggests that HAPAQ is a useful tool for retrospectively measuring PAEE in British adults. The correlations observed suggest that HAPAQ can accurately rank individuals within cohorts according to their total PAEE and time spent in VPA. This is important when exploring associations between PAEE and chronic diseases which have long latency periods.

## Abbreviations

BMI: (Body mass index); DIY: (Do it yourself); EE: (Energy expenditure); ICC: (Intraclass correlation coefficient); IQR: (Interquartile range); HAPAQ: (Historical adulthood physical activity questionnaire); HR: (Heart rate); MET: (Metabolic equivalent of task); MRC: (Medical Research Council); PA: (Physical activity); PAEE: (Physical activity energy expenditure); VPA: (Vigorous physical activity).

## Competing interests

The authors declare that they have no competing interests.

## Authors' contributions

Drafting/revising manuscript for important intellectual content: all authors

Final manuscript approval: all authors

## Supplementary Material

Additional file 1 **The HAPAQ questionnaire**. A sample of the HAPAQ questionnaire Click here for file
