# Supplementary material for: Validation of the historical adulthood physical activity questionnaire (HAPAQ) against objective measurements of physical activity
Source: Int J Behav Nutr Phys Act. 2010 Jun 24;7:54. doi: 10.1186/1479-5868-7-54 (PMC2902409; doi:10.1186/1479-5868-7-54)
Supplement: Additional file 1 — The HAPAQ questionnaire. A sample of the HAPAQ questionnaire [file 1479-5868-7-54-S1.DOC]

**Life Calendar**

Similar to timelines, life calendars are used to document events in your life as they occurred in time. Research has shown that when questions are asked about events and activities from the past, life calendars can be very useful in helping you to remember these events more accurately.

Our questionnaire will ask you about physical activity performed from the age of 20 years onwards. Therefore, to help you recall events, we will construct your own life calendar. To do this we would like you to try and remember the year of certain important life events, both happy and sad. We will ask you a set of questions to guide you, but you do not have to answer them if you feel uncomfortable.

We will then use the calendar to refer back to whilst filling in the questionnaire. However, the details you give here will not be used as part of the research project and at the end of the interview the calendar will be yours to keep.

All information you provide will be treated as confidential information.

To know when to start your life calendar, we need to know:-

1. What is your date of birth?....................................................................
2. What was the year was when you were 20 years old?................................

**Life Calendar questions**

**Housing**

1. What year did you buy your first house?
2. Do you remember any other house moves or changes in location as particularly memorable?

**Education and Work**

1. Have you been a student at anytime from the age of 20 years old?
2. Do you have any memorable job or career changes from the age of 20 years old?

**Life Events**

1. If you are married, what year did you get married?
2. If you have children, what year were they born?
3. If you have grandchildren, what year were they born?
4. Do you remember any significant deaths in your family or close friends?
5. Have you ever been divorced?

**Health**

1. Do you remember any particular time when you were admitted to hospital or had a long period of time off work due to illness?
2. Have you had any significant accidents or injuries that you remember?

**Holidays and Travel**

1. Are there any holidays you consider particularly memorable?
2. Have you ever spent a longer period of time travelling abroad or around the country?

**Others**

1. Can you recall any other memorable events in your life, such as passing a test/exam or winning the lottery?

***Sample of life calendar: Decade 80 - 89***

| **YEAR** | **World Events** | **Housing** | **Education/Work** | **Life Events** | **Health** | **Travel** | **Others** |
| --- | --- | --- | --- | --- | --- | --- | --- |
| **1980** | John Lennon shot  Ronald Regan became president |  |  |  |  |  |  |
| 1981 | Diana and Charles wedding |  |  |  |  |  |  |
| 1982 | Falklands war |  |  |  |  |  |  |
| 1983 |  |  |  |  |  |  |  |
| 1984 | World Aid following drought in Ethiopia |  |  |  |  |  |  |
| **1985** |  |  |  |  |  |  |  |
| 1986 |  |  |  |  |  |  |  |
| 1987 | Great storm 1987  Black Monday |  |  |  |  |  |  |
| 1988 | Lockerbie Airline bomb |  |  |  |  |  |  |
| 1989 | Berlin wall destroyed |  |  |  |  |  |  |

**Historical Adulthood Physical Activity Questionnaire (HAPAQ)**

***Sample: Period between 40 and 49 years old***

Activity in and around the house

Between the ages of 40 and 49 years old, how many hours on average per day did you spend sitting at home? This includes activities such as eating, drinking, reading, doing needlework, listening to radio, watching TV. You should give a separate answer for working and non-working days.

| *Hours per working day* | | | | | |
| --- | --- | --- | --- | --- | --- |
| ***Up to 1*** | ***Up to 2*** | ***Up to 3*** | ***Up to 4*** | ***Up to 6*** | ***Over 6*** |
| 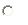 | 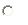 | 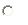 | 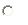 | 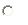 | 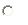 |

| *Hours per non-working day* | | | | | |
| --- | --- | --- | --- | --- | --- |
| ***Up to 1*** | ***Up to 2*** | ***Up to 3*** | ***Up to 4*** | ***Up to 6*** | ***Over 6*** |
| 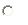 | 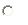 | 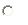 | 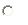 | 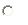 | 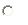 |

Did you do any **regular housework**? This includes preparing food, cooking, washing up, cleaning the house, doing the laundry and ironing. This should be on average at least one hour per day for at least one year.

| ***Yes*** | 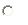 | ***No*** | 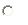 |
| --- | --- | --- | --- |

For how many years? 

| ***Years*** |  |
| --- | --- |

Did you do any **regular DIY or house and car maintenance** (excluding gardening)? This should be on average at least one hour per week for at least one year.

| ***Yes*** | 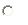 | ***No*** | 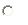 |
| --- | --- | --- | --- |

For how many years? 

| ***Years*** |  |
| --- | --- |

Did you do any **regular gardening**? This includes mowing, watering, weeding, pruning, digging, chopping wood. This should be on average at least one hour per week for at least one growing season.

| ***Yes*** | 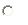 | ***No*** | 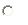 |
| --- | --- | --- | --- |

For how many growing seasons?

| ***Years*** |  |
| --- | --- |

Excluding travel to and from work, what **form of transportation** did you use most often **to get about**? You can give more than one form of transport if you think you used them equally as much.

| *Usual mode of travel* | | | |
| --- | --- | --- | --- |
| ***Car / motor vehicle*** | ***Walk*** | ***Public transport*** | ***Cycle*** |
|  |  |  |  |

Activity at work

Between the ages of 40 and 49 years old, how many years were you in each of the following occupations?

|  | ***Years*** |
| --- | --- |
| ***Student*** |  |
| ***Employed*** *(Paid and unpaid: voluntary work)* |  |
| ***Housewife/husband*** |  |
| ***Retired*** |  |
| ***Professional sport person*** |  |

Guide: The sum of years should not be over 10, unless the participant has been carrying 2 occupations the same time.

I am now going to show you some pictures which demonstrate four different types of work classified depending on the intensity of physical activity involved.

During this decade try to remember the type of activity your work involved, excluding housework. Try to classify each job into one of these four categories, which ever is most representative of what you did.

For each category, how many years of the decade did you hold such a job and how many hours per week on average did you work?

|  | ***Years*** | ***Months*** | ***Hours per week*** |
| --- | --- | --- | --- |
| ***Sedentary occupation*** |  |  |  |
| ***Standing occupation*** |  |  |  |
| ***Manual work*** |  |  |  |
| ***Heavy manual work*** |  |  |  |

Guide: The sum of years should not be over the number of years previously indicated unless some works have been held concomitantly.

For how many years did you work as a **professional sports person**? On average how many hours per week did you do this job? What sport was it?

|  | ***Years*** | ***Months*** | ***Hours per week*** |
| --- | --- | --- | --- |
| ***Sport:*** |  |  |  |

Commuting to work

Between the ages of 40 and 49 years old, regarding travel to and from your place of work or study, did you ever **regularly** travel **by bike**, at least once a week for at least one year?

| ***Yes*** | 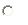 | ***No*** | 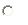 |
| --- | --- | --- | --- |

For how many years did you do this? 

| ***Years*** |  |
| --- | --- |

On average, how many journeys per week did you cycle, counting return journeys only? 

| ***Number of journeys*** |  |
| --- | --- |

On average, how many miles was that return journey?

| ***Mileage*** |  |
| --- | --- |

Did you ever **regularly** travel to your place of work or study **by foot**, at least once a week for at least one year?

| ***Yes*** | 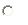 | ***No*** | 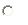 |
| --- | --- | --- | --- |

For how many years did you do this?

| ***Number of journeys*** |  |
| --- | --- |

On average, how many journeys per week did you walk, counting return journeys only? On average, how many miles was that return journey?

| ***Mileage*** |  |
| --- | --- |

Sporting and Recreational Activity

From the ages of 40 to 49 years old, did you do any **regular sports in a competitive or strenuous nature**? By this we mean it made you out of breath and/or sweat a lot. It must have been undertaken for at least one hour per week for at least one year.

| ***Yes*** | 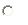 | ***No*** | 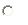 |
| --- | --- | --- | --- |

I am now going to show you some illustrations of different sports which may be undertaken on a competitive level.

Can you identify your sport amongst these? If not what other sports did you do between the ages of 40 and 49 years old?

For how many years did you do this sport and on average how many hours per week?

|  | ***Years*** | ***Hours per week*** | ***Minutes per week*** |
| --- | --- | --- | --- |
| ***Swimming*** |  |  |  |
| ***Cycling*** |  |  |  |
| ***Running*** |  |  |  |
| ***Football, rugby or hockey*** |  |  |  |
| ***Cricket*** |  |  |  |
| ***Volleyball, basketball or netball*** |  |  |  |
| ***Racket sports*** |  |  |  |
| ***Aerobics*** |  |  |  |
| ***Fighting sports*** |  |  |  |
| ***Other sport:*** |  |  |  |
| ***Other sport:*** |  |  |  |

From the ages of 40 to 49 years old, did you undertake any **regular sport or recreational exercise on a casual basis**? This means it was not competitive and didn't make you out of breath and/or sweat a lot. It must have been undertaken for at least one hour per week for at least one year.

| ***Yes*** | 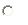 | ***No*** | 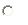 |
| --- | --- | --- | --- |

I am now going to show you some illustrations of different sports which may be undertaken on a competitive level.

Can you identify your sport amongst these? If not what other sports did you do between the ages of 40 and 49 years old?

For how many years did you do this sport and on average how many hours per week?

|  | ***Years*** | ***Hours per week*** | ***Minutes per week*** |
| --- | --- | --- | --- |
| ***Walking for pleasure*** |  |  |  |
| ***Swimming for pleasure*** |  |  |  |
| ***Jogging*** |  |  |  |
| ***Conditioning exercises*** *(Yoga, weights…)* |  |  |  |
| ***Golf*** |  |  |  |
| ***Bowling*** |  |  |  |
| ***Fishing or hunting*** |  |  |  |
| ***Other exercise undertaken on a casual basis:*** |  |  |  |
| ***Other exercise undertaken on a casual basis:*** |  |  |  |

***Prompt cards***

**OCCUPATIONS**

| 1. Sedentary occupation   **You spent most of your time sitting (such as in an**  **office).** |  |
| --- | --- |
| 1. Standing occupation   **You spent most of your time standing or walking.**  **However, your work does not require intense physical**  effort (e.g. shop assistant, hairdresser, guard). |  |
| 1. **Manual work**   **This involved some physical effort including handling of**  **heavy objects and use of tools (e.g. plumber,**  **electrician, carpenter).** |  |
| 1. **Heavy manual work**  **This implied very vigorous physical activity including** **handling of very heavy objects (e.g. dock worker, miner,** **bricklayer, construction worker).** |  |

SPORTS

| 1. Swimming |  |
| --- | --- |
| 2. Cycling |  |
| 3. Running |  |
| 4.Football, rugby or hockey |  |
| 5. Cricket |  |
| 6. Volleyball, basketball  or netball |  |
| 7. Racket sports |  |
| 8. Aerobics |  |
| 9. Fighting sports |  |
| 10. Other sports |  |

**EXERCISES**

| 1. Walking for pleasure |  |
| --- | --- |
| 2. Swimming for Pleasure |  |
| 3. Jogging | 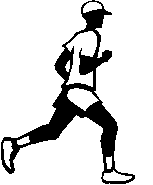 |
| 4. Conditioning exercises (e.g. yoga, weights etc) |  |
| 5. Golf |  |
| 6. Bowling |  |
| 7. Fishing or Hunting |  |
| 8. Other exercises |  |
